# Research in minors with a recent history of trauma—A bold venture?

**DOI:** 10.1007/s40211-019-00328-7

**Published:** 2020-01-30

**Authors:** Doris Mayerhofer, Julia Schwarzenberg, Regina Rüsch, Gertrude Bogyi, Katrin Skala

**Affiliations:** 1 Department of Child and Adolescent Psychiatry, Medical University of Vienna, Waehringer Guertel 18–20, 1090 Vienna, Austria; 2 Ambulatorium “Die Boje”, Hernalser Hauptstraße 15, 1170 Vienna, Austria

**Keywords:** Trauma, Minors, Research, Barriers, Trauma, Kinder, Jugendliche, Rekrutierung

## Abstract

Little research has been performed so far on the mental health state of grieving and recently traumatized children. “The Buoy” (“Die Boje”), a low threshold ambulatory provides non-bureaucratic help and short time psychotherapy to children and adolescents in need of professional support at no charge and treats about 1400 minors per year. Whilst performing a study on these patients with special regard to their social network, we found the process of recruitment to be extraordinarily challenging. Only about 25% of the eligible patients could be recruited successfully within during the period of one year. In this paper we try to examine the barriers we had to overcome in gaining access to the sensitive field of grieving and traumatized children and adolescents who rely on low threshold psychotherapeutic and neuropsychiatric support and analyze the factors leading to the high number of dropouts. In addition, the consequences for our results will be discussed.

## Zusammenfassung

Bis dato gibt es nur wenige Studien, die sich mit der psychischen Gesundheit von rezent traumatisierten und trauernden Kindern auseinandersetzen. „Die Boje“ ist ein niedrigschwelliges Ambulatorium, welches rund 1400 Kindern und Jugendlichen, die kostenfrei professionelle Hilfe benötigen, unbürokratisch Kurzzeitpsychotherapie anbietet. Im Rahmen einer Studie an diesen Kindern und deren sozialem Netzwerk stellte sich die Rekrutierung als außergewöhnlich schwierig dar. Nur 25 % der infrage kommenden PatientInnen konnten innerhalb des ersten Jahres rekrutiert werden. In dieser Arbeit untersuchen wir die Gründe für die Schwierigkeiten, Zugang zu dieser Klientel zu bekommen, und analysieren und diskutieren die Faktoren, die zu der hohen Zahl an Drop-Outs geführt haben.

## Introduction—Problems?

Research on potentially traumatized and grieving individuals investigating their state of mental health as well as risk- and protective factors is a challenging topic. The recruitment of persons who recently encountered adversities requires tact, empathy and special caution to their specific circumstances. If the subjects in question are children with a history of adverse childhood experiences (ACEs), the course of action necessitates even more carefulness regarding their vulnerability. Nevertheless, it is important to get access to the field of potentially traumatized children and assess crucial aspects of their everyday lives to learn how they can be supported in a more effective way. Investigating them as short as possible after the traumatic event constitutes a very challenging, yet highly relevant issue. By exploring primary healthy children after having experienced a traumatic event but before trauma sequels develop it should be possible to better delineate resilience factors from coping mechanisms.

However, based on prior research suggesting that an individual’s personal ties may contribute to their resistance against stresses and strains [1, 3, 5, 9] we set out to confirm this hypothesis conducting a prospective study, intended to run from January 2018 until December 2020. We recruit our sample at the low-threshold ambulatory “The Buoy” (Ambulatorium “Die Boje”, www.die-boje.at) situated in the 17th district of Vienna, Austria. The examination of our subject’s mental health shortly after experiencing an adverse event with special regard to the grown-up attachment figures is defined as our main matter of interest. We monitor participants with a history of loss, violence or divorce and aim to assess symptoms of depression, anxiety or posttraumatic stress.

As we depend on own resources, the study site’s and the study populations’ options it was clear that certain obstacles must be assumed in the attempt to investigate resilience in children who rely on psychotherapy in a low-threshold outpatient setting. Looking through studies conducted on traumatized children we found that there are hardly any descriptions of recruitment processes and potential barriers researchers had to face.

Literature reviews quite sufficiently sum up described recruitment strategies for children in different matters of research like behavioral health (REACH-Strategies, [6]) or pediatric dental health [8]. Nevertheless, only some of the mentioned strategies apply to our sensitive field of investigation.

However, even the best recruitment strategies are likely to fail if there are factors which hinder a family’s participation. Thus, besides functional recruitment strategies, we must take note of the barriers which occur throughout recruitment processes. Sifers, Puddy, Warren and Roberts [7] conducted a literature review on 260 papers of four journals publishing in the field of child psychology and conclude that most papers hold no information about the drop-out rates of eligible participants or the attrition of study samples.

In their survey, Rau et al. [4] examined potential barriers that must be overcome in running studies in organizations of interest. Overstress, lack of time and non-acceptance towards the study or the researcher or fear of additional efforts have prior been described as reasons why organizations rejected participation in research projects. They also criticize the lack of systematic description on the barriers of recruitment, especially in scientific research on children in sensitive issues like sexual abuse. They found that gaining access to the organizations of interest is one major barrier.

In our case, we corporate with our organization of interest and luckily, we are very well being supported by the ambulatory’s team, as they provide all the resources which are necessary to conduct a study and the they support the process of recruitment. Still, we struggle with other obstacles in the recruitment of subjects. Wondering about the low number of participants due to the high number of potential subjects we examined the attrition of our sample. This short descriptive report provides recruitment data and discusses the barriers we had to overcome in the recruitment of grieving and traumatized children as outpatients in a low threshold setting during the first year of the study and discuss our recruitment opportunities.

## Method

### Participants and procedure

#### Eligible patients

**Fig. 1:**
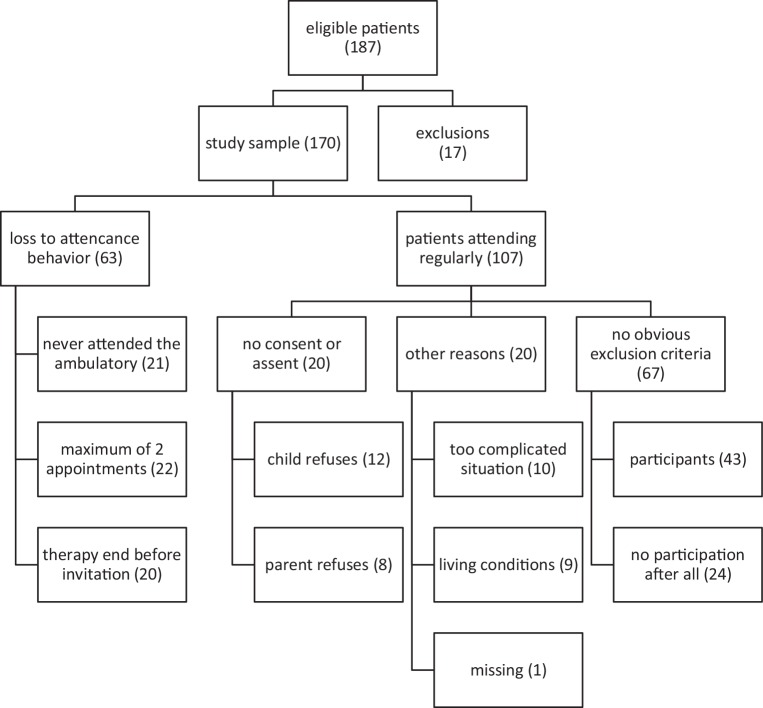
Path of eligible patients throughout the recruitment process

The inclusion criteria comprise children aged 8 to 12 calling on the ambulatory “The Buoy” with a history of loss, violence or divorce. A total of 177 children who called on “The Buoy” from January through December 2018 met the inclusion criteria. Ten patients were added to the preselected cases. They did not seem to fit at first sight, or they already have been patients before the study started. We however decided to invite them as their psychotherapists considered them to fit the study’s requirements. By the end of the study’s first year, 36 participants completed the investigation or at least started working on the questionnaires. Seven more of the eligible patients from 2018 took part in the beginning of 2019, so we added their descriptive data to our results (see Fig. 1).

##### Requests for therapy

The reasons why children and their families seek help at the ambulatory are heterogeneous and some families are strained by multiple problems. For an overview we combined the subjects into different groups: sudden death of a loved one, anticipated death of a loved one, life threatening condition of a loved one, relative afflicted by a physical or mental illness, experiencing physical violence, witnessing (or learning from) physical violence against a loved one, emotional violence, flight, acrimonious divorce or separation and other reasons (e.g. patient suffering from school or behavioral problems). If a child is confronted with two or more problems, we based the allocation on the one reason that led the patient’s family to call for help. As they called for help, 58 families reported two and 13 families even three different problems they had to deal with at the same time (Fig. 1).

**Fig. 2:**
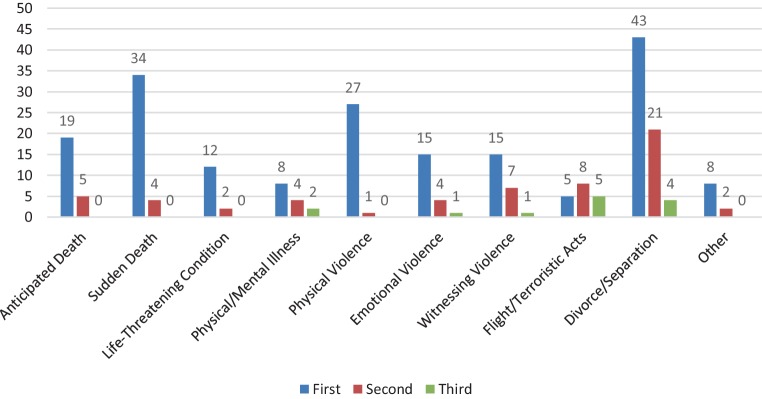
Frequencies of the different requests for therapy and accumulation of risk factors

Table 1 summarizes the therapy requests of our 187 eligible patients. It shows that 35% were confronted with the death or a life threatening condition of a loved one, 34% witnessed or experienced any kind of violence, 4% deal with a chronic mental or physical illness or any other impairing condition of a loved one, 23% of the sampled patients are children who experienced traumatic divorce and 4% are allocated in the group “other reasons”, these children report school or behavior problems or suffer from mental disorders like depression or anxiety disorders (see Fig. 2).

**Table 1:** Sample Characteristics and frequency of requests for therapy comparing non-participants and participants

	Eligible Patients (*n* = 187)	Participants (*n* = 43)
**Sample Characteristics**		
*Mean Age*	10.15	10.37
*Sex*		
Female	*n* = 107	*n* = 21
Male	*n* = 76	*n* = 22
No information	*n* = 4^a^	–
**Therapy requests**	***n*** **(%)**	***n*** **(%)**
*Loss*	*65 (35%)*	*17 (39%)*
Sudden death	34 (18%)	4 (9%)
Anticipated death	19 (10%)	8 (18%)
Life threatening condition	12 (7%)	5 (12%)
*Chronic/severe illness*	*8 (4%)*	***–***
*Violence*	*62 (34%)*	*18 (42%)*
Physical violence	27 (15%)	5 (12%)
Emotional violence	15 (8%)	4 (9%)
Witnessing violence	15 (8%)	7 (16%)
Flight	5 (3%)	2 (5%)
*Divorce*	*43 (23%)*	*8 (19%)*
*Other*	*8 (4%)*	*–*

Table 1 shows the sample characteristics and the distribution of therapy requests in all eligible patients who called on the ambulatory between January and December 2018, the left column describes all screened eligible patients, the left column indicates the data of the participants

^a^Four children fit the study requirements but never attended the ambulatory. Due to the information received from the ambulatory’s casebook we do not know the sex of these three children. In one child, who never attended the ambulatory, the therapy request is unknown

### Study site

To test our hypotheses, we recruit our participants at the low threshold ambulatory “The Buoy”. The ambulatory was founded in 2002 and is situated in the 17th district of Vienna, Austria. “The Buoy” supports children and adolescents who are confronted with different adversities like the loss or severe illness of a loved one, the experience physical or emotional violence as well as divorce. Contracts with health insurance groups and donations enable the institution to support children, adolescents and families at no charge. The psychotherapists, psychologists and psychiatrists who work there offer crisis intervention, brief psychotherapeutic and neuropsychiatric intervention, group therapy as well as psychological diagnostics. The families and legal guardians can also receive counseling.

According to the ambulatory’s annual report from 2017, 1420 patients received help at the ambulatory throughout the year. The main reasons for attending the ambulatory were the sudden or anticipated loss of a loved one (19.5%), having a mentally or physically ill relative (16.5%), flight (18.2%) and divorce or separation (23.3%). The ambulatory’s unique specialization as well as the ability to support non-bureaucratically and free of cost render “The Buoy” the place to go for youngsters in need of professional psychotherapeutic support. Due to the low-threshold and cost-free offer we assume that the ambulatory’s clientele faces additional strains despite their request for therapy.

### Recruitment

Concerning the patients’ vulnerability, the process of recruitment requires special caution. Thus, the recruitment starts at the registry. Families in need usually fix their first appointment by phone. The secretaries arrange the first appointment and if a potential patient seems to fulfill the inclusion criteria at first sight, they add a “study document” to the health record which fosters the communication between the ambulatory’s team and the prior researcher. Hence, all registered cases who fit the study’s requirements at first sight are considered being eligible. Within the first therapy sessions, the psychotherapists check if the inclusion criteria are met and if a child’s mental state allows participation. If so, the psychotherapist informs the legal guardians and the patients about the ongoing study and its aim and invites them to participate. The psychotherapist notes if the family accepts or refuses participation and hands the document to the prior researcher. Only if both, the child and the guardian, agree participation the prior researcher can call the family to fix an appointment. At the invitation call the prior researcher briefs the guardians about the study’s aims and frame conditions, such as the planned duration. Since we recruit outpatients, flexibility and adjustment to the families’ needs are self-evidently major accommodations the prior researcher must concede.

## Results

The presented descriptive data and graphics sum up the attempts of the study’s first year and give an overview of the recruitment process. Within the first year, 187 children were registered at the ambulatory and were considered being eligible in a view of the studies inclusion criteria, but in the end only 43 participants could be recruited. Fig. 1 shows the path of eligible subjects through the process of recruitment and gives an overview of the major problems we had to face.

### Attendance behavior (*N* = 63)

We lost 63 patients before they could even be asked to participate.

*Never came*. Twenty-one children were registered at the ambulatory but never attended “The Buoy” after all.

*Maximum of two appointments*. Further 22 patients had a maximum of 2 therapy sessions. Putative reasons are therapy break ups or the family attended the ambulatory only in need for counseling.

*Therapy end.* In 20 cases patients were not invited to participate due to therapy break up or therapy end, if only a few sessions of psychotherapeutic counseling satisfied the needs of the child.

### Other reasons (*n* = 20)

If the familial situation turned out to be very complex and straining (*n* = 10) we refrained from making contact for the time being. In 9 cases, the living conditions hindered the participation. Only in 1 eligible subject the reason for dropping out is unknown.

### No consent or assent (*N* = 20)

Refusal accounts only for 11% of the drop outs, 8 guardians and 12 children clearly retained.

### No obvious exclusion criteria (*N* = 67)

Only 43 subjects who registered between January and December could be recruited successfully. In 24 cases the appointment could not be fixed as the families did not show up as agreed upon, they did not find the time to participate, they showed little compliance, they delayed the appointment several times or there were not contactable anymore.

### Exclusions (*N* = 17)

We excluded eligible patients if exclusion criteria were fulfilled or if a child did not consult the ambulatory to receive psychotherapeutic help or did not meet the study requirements.

### Sample characteristics

The average age of our sample is 10.37 and the gender distribution is balanced (21 females, 22 males). Out of 43 participants, 29 live in a single parent household due to divorce or death of a parent. 27 children have a migration background, 6 were born abroad, 5 of them are refugees. Thus, we can assume that the bigger part our participants and their family members are exposed to accumulative risk factors (see Table 1 for differences between participants and non-participants).

## Discussion

Studying the participation of children and adolescents in scientific research is an unspectacular but yet crucial field of research aiming to identify and systematically describe potential sources of bias. Looking at all our data, the most prominent question arising is whether other authors struggle with similar problems. Studies on childhood trauma often only report successfully recruited subjects. Thus, we want to discuss our experiences of research within a sensitive and rather dynamic population.

### Barriers related to the study population

Our data clearly points out that the patient’s attendance behavior accounts to the high number of drop outs in our outpatient population. More than a third of the eligible patients dropped out before they could even be asked to participate. In our age range of interest, 12% of the eligible patients did not show up to their first appointment without cancelling, 13% stopped attending the ambulatory after a maximum of 2 appointments and 11% stopped their therapy before they could be invited to participate. Thus, the fluctuation is one of the most important problems to deal with in the recruitment of an outpatient sample. Unfortunately, there is very little information about those who drop out. In the group of our non-participants we only know about the child’s age, sex and the requests for therapy, but we do not know why in the end they do not come to receive the support they have asked for. Furthermore, we have no information about the socioeconomic status, a potential migration background or their reasons for non-attendance. In a view of statistical purposes and the generalizability of our data, we must describe our recruited sample accurately to correctly apply the knowledge we derive from our participants.

Sixty-three of our remaining study sample met the inclusion criteria and agreed to an invitation call. Still, our efforts to invite them failed in 24 cases. Some families did not clearly refuse participation, but making contact was impossible, some showed little compliance as they did not show up as agreed upon or they delayed their appointments repeatedly. In their follow up, Jud, Lips and Landolt [2] made great efforts in contacting their participants, such as writing an invitation letter and attempting 5 consequent invitation calls. In the end they report a successful recruitment rate of 45% within their study sample. Our efforts were successful in only 25%. As our study relates to the ambulatory, we must take care that our bid for invitation does not provoke opposition in the outpatient’s families. Thus, after three unsuccessful attempts to call a family or to fix an appointment, we refrained from further invitation calls. In the first place, the families have the right to refuse participation without justification. Secondly, being too pushy would neither be in line with ethical considerations nor with the approach of “The Buoy”, since the clientele deserves protection from additional strains. Thus, we refrained from being too intrusive.

Further barriers related to the study population can be found within their everyday hazards. Some of the families mentioned their lack of time or the fact that they are being stressed out. We take note of the fact that participating in our study causes inconvenience and requires the child and the legal guardian to both take the time to come to “The Buoy” and stay for about 90 min. This leads us to one major barrier in the process of recruitment: research on children (especially as outpatients) necessitates the recruitment of both, the youngsters and their legal guardians. We deal with two different groups of persons with different needs. We must meet the desires of the parents’ work and family situation. In some cases, participating the study would have required absence from work, some have appointments for their therapy-sessions as well or want to use spare time for other activities they do not want to neglect. At “The Buoy” we are recruiting a population dealing with economic insecurity and at some points of time we are confronted with rather difficult familial situations, for instance when the guardian is a working single parent who has to take care of the participants’ siblings as well. Since our patients are outpatients, taking the time to participate the study turns out to be very difficult to a major part of our eligible patients, especially if the study appointment however could not be combined with the patient’s therapy sessions.

Regarding the children, we had to pay heed to different requirements at school and spare time activities like participating sports clubs or music lessons. Only in 6% the children showed no interest in participation in the study. The prior researcher tried to accommodate to the families’ time resources and needs with flexibility, offering appointments in the evening or combining the study appointments with their therapy sessions, but this offer turned out to be helpful only in some cases. Holidays could provide a favored time span to fix appointments, but in the end these appointments were also likely to be canceled or missed.

Finally, and contrary to other countries, financial or other rewards as a compensation for participation or adherence are not regarded with favor by most ethics committees; subjects that are on one hand children and on the other hand recently traumatized are hereby considered as members of a “particularly sensitive” group. Hence, no compensation for their efforts or time is possible. Under the given circumstances this might in fact be one of the major factors compromising recruitment and adherence in this study. Working in the field of a low threshold offer and recruiting families with multiple psychosocial risk factors even little rewards like vouchers could underline the value of their participation.

### Barriers related to the study site

We are lucky to corporate with the ambulatory “The Buoy” and enjoy the support provided by the team. Still, there are some obstacles which are related to the study site. The ambulatory is situated in the 17th district of Vienna, Austria. In a view to the study’s requirements, it is necessary that both, the child and the guardian, attend the ambulatory to sign the informed consent document and to answer the questionnaires. If combining the study appointment with the therapy sessions is not possible, we have difficulties to agree upon extra appointments, as the families must arrive at the ambulatory and time the appointment. To families living in outlying areas of Vienna, arriving at the ambulatory might take about an hour. Thus, the prior researcher self-evidently has to offer an appointment that suits the families’ desires. The opportunity to combine therapeutic sessions with the study appointments really plays to the needs of some families but requires the researcher’s and the ambulatory’s flexibility. “The Buoy’s” team is comprised by about 25 coworkers and all of them work there part-time. Besides providing enough space and time for the psychotherapists and their patients, making a room available to ensure the prior researcher space to hold study appointments is a logistic issue. “The Buoy” thankfully provides us all the resources we need to conduct the study. Still, there are phases throughout the week when space cannot be available.

## Conclusions

In contrast to the assumptions Rau et al. [4] draw from their research within sensitive populations, we enjoy the support received from our study site. The two major barriers we had to face were the attrition of our outpatient population as well as the psychosocial risk factors and the daily hazards our study sample must deal with.

Consequently, the question is how can we foster participation within this sensitive population? A promising factor facilitating recruitment can be found in shifting the focus from extrinsic motivational factors to the enhancement of intrinsic motivation regarding the participation. Concerning our current study, a families’ interest in matters of scientific research in general, feelings of social responsibility and the idea of contributing to the improvement of future treatment approaches seemed to play a superordinate role to both, the children’s and their guardians’ commitment.

Besides rewards, indirect benefits of participation seemed to foster compliance too. In most of the participating families, the guardians seemed to welcome the offer of an additional psychological assessment and feedback on behalf of their children. Subsequently, after finishing the study appointment children usually reported that they enjoyed the appointment and found it interesting to answer the questionnaire items. Some families seemed to accept the invitation as a token of our esteem to point to their daily life hazards.

## Limitations

Information about the age, sex and therapy request of our study sample is available, but we do not know about the parents’ working situation, the socioeconomic status, migration background or additional strains the families might deal with. Since a patient’s psychological assessment at the ambulatory is scheduled after approximately 30 units of crisis intervention, there is no quantitative data about the youngster’s mental health state in the beginning of their therapy. Thus, we cannot compare the data of those who participate to data of those who do not.

As Jud et al. [2] conclude, more research in the issues of recruitment is needed to assess variables which could bias the recruitment of participants, suggesting that the socioeconomic status could be of special interest. The researcher only gets into contact with the families if they agree to participate. Thus, we lack access to demographic data of the eligible patients.

## Implications

In a synopsis of our data the question arises which motivational aspects influence the attendance behavior and the readiness to participate in scientific research of families in need of professional support free of charge. Future research should pay attention to the examination of motives and expectations on the part of children and guardians towards participating in scientific studies. Furthermore, recruitment bias could be a more common phenomenon than expected, so description of the characteristics of both, participants and non-participants, could help to correctly apply the knowledge in future studies.
